# Predictive models for post-ERCP pancreatitis: a systematic review and meta-analysis

**DOI:** 10.3389/fgstr.2025.1629698

**Published:** 2026-03-05

**Authors:** Zhihang Zhong, Li Liu, Jia Liu, Qin Xie, Jing Wu

**Affiliations:** 1 Department of Gastroenterology, Southwest Hospital, Army Medical University, Chongqing, China; 2 School of Nursing, Army Medical University/Third Military Medical University, Chongqing, China; 3 Department of Hepatobiliary Surgery, Southwest Hospital, Army Medical University, Chongqing, China

**Keywords:** Post-ERCP pancreatitis, predictive models, meta-analysis, AUC, model performance

## Abstract

**Background and aims:**

Post-ERCP pancreatitis (PEP) is the most common complication following ERCP, leading to significant clinical and economic consequences. Predictive models for PEP can help identify high-risk patients and guide preventive strategies. However, the performance of these models varies, and a comprehensive evaluation is lacking. This study aims to assess the accuracy, reliability, and risk of bias in existing predictive models for PEP.

**Methods:**

A comprehensive search was conducted across five databases (PubMed, Embase, Web of Science, Cochrane Library, and CNKI) for studies published until January 2025. Studies that developed or validated predictive models for PEP were included. Models with external validation sets were included in a meta-analysis. Model performance was assessed using the area under the receiver operating characteristic curve (AUC), sensitivity, specificity, and calibration. A random-effects meta-analysis was performed, with heterogeneity assessed using I² statistics. Data extraction and risk of bias were conducted using a standardized template combining the CHARMS and PROBAST tools.

**Results:**

Twenty-three studies (21 model development studies and 2 external validation studies) were included, presenting 21 predictive models for PEP. Nine models incorporated external validation, with one study recalibrating an existing model and another externally validating two prior models. The mean events per variable (EPV) across studies was 10.2 (2.2 to 22.4). The pooled AUC for externally validated models was 0.79 (95% CI: 0.75–0.83). Machine learning models demonstrated higher AUC (0.84) than traditional logistic regression models (0.76). Common predictive factors included difficult cannulation, female sex, pancreatic duct dilation, and a history of pancreatitis.

**Conclusions:**

Predictive models for PEP show potential for improving patient risk stratification. However, variability in model performance, lack of external validation, and significant bias in many studies limit their clinical applicability. Further external validation, model refinement, and improved bias control are essential for broader clinical implementation.

**Systematic Review Registration:**

https://www.crd.york.ac.uk/PROSPERO/view/CRD42024626168, identifier CRD42024626168.

## Background

Post-ERCP pancreatitis (PEP) is the most common complication after undergoing endoscopic retrograde cholangiopancreatography (ERCP). The incidence of PEP varies from 3.5%–9.7% in average-risk procedures to 14.7% in high-risk procedures (1, 2). Patient-related and procedural factors have been identified as key factors influencing the development of PEP (2–4).

Meta-analyses have highlighted certain factors that significantly increase the risk of PEP, such as female sex, having a history of acute pancreatitis, older age, undergoing precut sphincterotomy, having pancreatic duct injection, and difficult cannulation (5–7). New ERCP techniques, such as pancreatic sphincterotomy, precut sphincterotomy, and the Double-Guidewire Technique(DGT), have shown the potential to reduce the incidence of PEP (8, 9). The endoscopist’s skill level, the medical institution’s size, and the procedural volume also play significant roles in PEP occurrence (10–12).

Despite knowledge of these factors, PEP remains unpredictable. Recent studies have developed predictive models using traditional statistical approaches and machine learning techniques, presented in nomograms, scoring systems, and online calculators (11–14). The Pancreatitis Risk Score (PRS) proposed by the American Society for Gastrointestinal Endoscopy (ASGE) incorporates age, female sex, and pancreatic ductal dilatation, achieving 80% sensitivity and 70% specificity (13). Machine learning has enabled the development of more sophisticated models capable of capturing complex, nonlinear relationships (14). Brenner et al. (2025) developed a gradient-boosted machine model using data from 12 trials, achieving an AUC of 0.70 in validation and 0.74 in a prospective study (15).

Some models have shown good performance. However, their predictive accuracy varies significantly across studies and patient populations (3). No model has been widely implemented. Few models have undergone rigorous external validation, and direct comparisons in multicenter studies remain scarce, limiting their clinical applicability.

This study systematically reviews all published PEP prediction models. We evaluate their methodological frameworks, predictor variables, and performance metrics, including discrimination, calibration, and validation strategies. Additionally, we compare predictive accuracy across models and assess their robustness and clinical applicability.

## Methods

This study followed the PRISMA guidelines (16), ensuring compliance with eligibility criteria, execution of a comprehensive search strategy, study selection process, data extraction, risk-of-bias evaluation, and data analysis. The protocol was registered in PROSPERO (CRD42024626168).

### Search strategy

A comprehensive search was conducted in five databases: PubMed, Embase, Web of Science, the Cochrane Library, and CNKI, with a search to January 1, 2025. The search strategy for PubMed was as follows:

1. #1 “ERCP” OR “endoscopic retrograde cholangiopancreatography”
2. #2 “post-ERCP pancreatitis” OR “PEP” OR “pancreatitis after ERCP” OR “ERCP complications”
3. #3 “prediction model” OR “predictive model” OR “predict*” OR “prognostic model” OR “risk assessment” OR “risk stratification” OR “clinical prediction tool”
4. #4 “sensitivity” OR “specificity” OR “AUC” OR “area under the curve” OR “ROC curve” OR “calibration” OR “discrimination” OR “decision curve analysis”
5. #5 #1 AND #2 AND (#3 OR #4).

Appendix 1 contains detailed search strategies for 5 databases.

### Inclusion and exclusion criteria

We included studies on developing and validating predictive models, regardless of whether external validation was performed.

The inclusion criteria were based on the key elements of the PICOT system. The PICOTS statement was as follows:

1. P (Population): Patients aged 18 years or older who underwent ERCP.
2. I (Index model): Models used to predict Post-ERCP Pancreatitis (PEP).
3. C (Comparator model): Not applicable.
4. O (Outcome): The outcome was defined as the occurrence of PEP. We accepted all diagnostic criteria adopted by the studies.
5. T (Timing): Models based on pre-ERCP or intraoperative data were used to predict PEP. The timing of PEP occurrence was defined according to the diagnostic criteria reported in the studies.
6. S (Setting): Studies conducted in any medical setting, including single-center, multicenter, and international multicenter studies, without restrictions on the level or geographic location of the healthcare institution.

We excluded studies from which model performance metrics could not be extracted, studies without full text available, and studies published as letters, protocols, reviews, or case reports. Study selection and data extraction.

All search results were imported into Zotero for merging and deduplication. Two evidence-based trained reviewers (ZZH and LJ) screened the titles and abstracts, obtaining the full text of potentially eligible studies. The two reviewers independently assessed whether the studies met the inclusion criteria, with any disagreements resolved through discussion. Discrepancies were resolved by a third reviewer when necessary.

### Risk of bias assessment

We used a standardized Excel template developed by Borja M. Fernandez Felix (17) to extract data and assess the risk of bias and applicability of the predictive model studies. This template combines the CHARMS (17) and PROBAST (18) tools into one file, streamlining and standardizing data extraction and bias risk assessment, reducing error risk, and improving reviewers’ consistency. The CHARMS worksheet follows Moons et al.’s framework (18, 19). The data extraction table was built around 11 CHARMS domains. The extracted data included study characteristics (author, publication year, title, model name), study design (data source, recruitment methods, study setting, number of centers, inclusion/exclusion criteria, participant characteristics such as age and gender), outcomes, predictors, data characteristics (sample size, events, EPV, missing data handling), model development, performance, validation, and final model details.

Reviewers filled in all highlighted cells and used dropdown menus or free text responses. If information was unavailable, reviewers indicated “NA.” The PROBAST tool assessed bias and applicability across four domains: participants, predictors, outcomes, and analysis. Bias risk was rated as low, high, or unclear.

### Statistical analysis

Studies were compared based on their characteristics. Research characteristics, quality, and model performance data were tabulated. Descriptive statistics summarize study types, outcomes, statistical methods, and predictors. A meta-analysis of externally validated predictive models was performed using Stata 18.0, with the primary effect size being the area under the receiver operating characteristic curve (AUC). The standard error (SE) for each study’s AUC was calculated based on sample size and AUC using the formula (20) $SE=\sqrt{AUC\times (1-AUC)/n}$, The data were declared using the meta set command, with the effect size being AUC and the standard error pre-calculated. A random-effects model was used for the meta-analysis, and heterogeneity was assessed using I².

## Results

**Figure 1:**
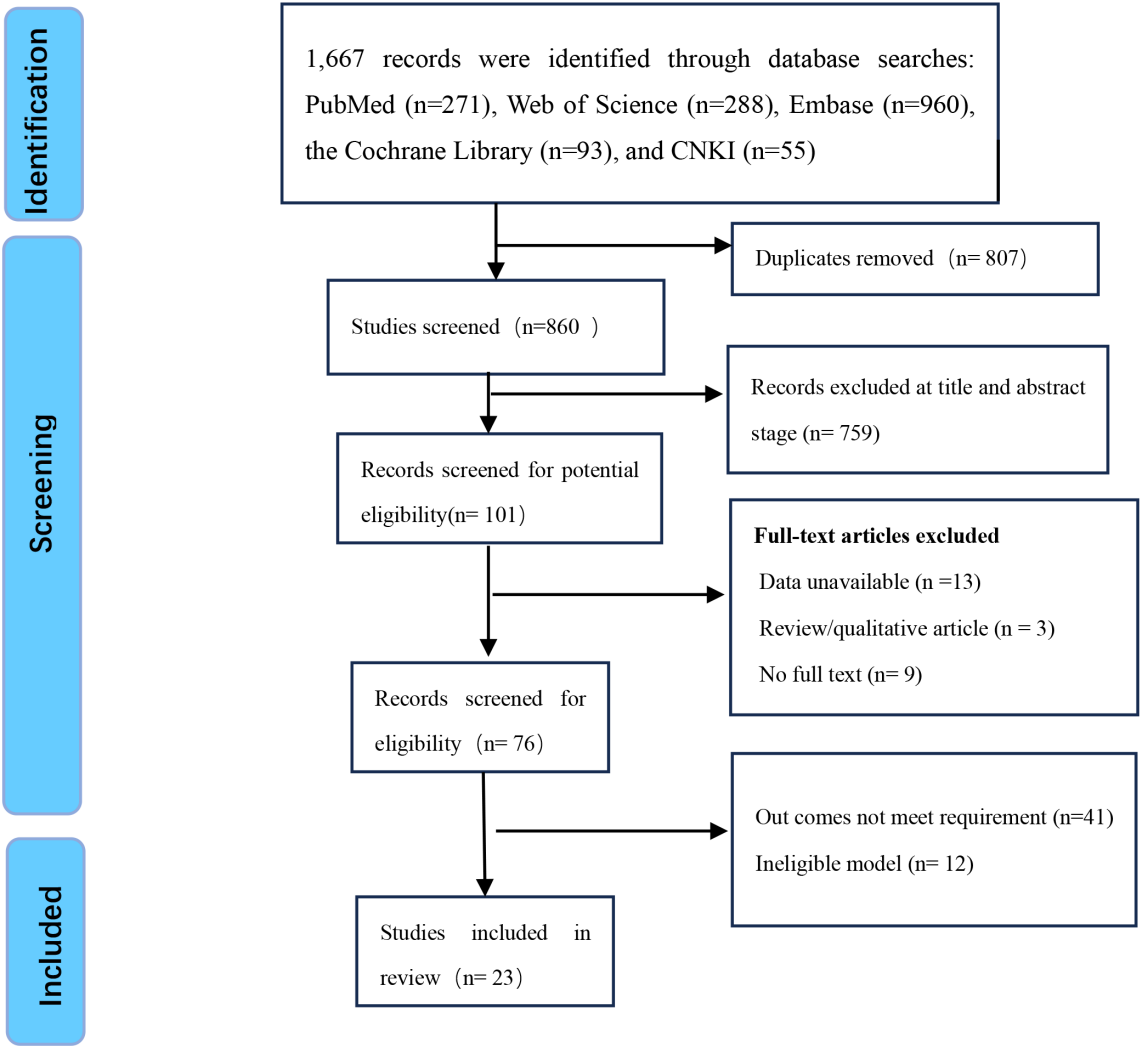
PRISMA flowchart for our study’s identification and selection.

A total of 1,667 relevant articles were identified. After removing 807 duplicates, 76 articles were screened based on titles and abstracts, and a full-text review was conducted. Ultimately, 23 articles were included in the study (**Figure 1**).

### Study characteristics

**Table 1** summarizes the characteristics of the included studies, including author, year, study design, enrolment period, study setting, study region, and participant demographics. Among the 23 included studies, 6 (9, 33, 35–38) focused exclusively on model development, 7 (21–23, 28, 29, 31, 32)on model development and internal validation, 7 (24–27, 30, 34, 39)on model development with both internal and external validation, and 2 (41, 42) on external validation alone. Notably, one study (41) that focused solely on external validation also recalibrated the original model (35) and modified its predictive factors.

**Table 1:** Characteristics of the studies included in the systematic review.

Author, year	Study design	Enrolment period	Study setting	Study region	Participant characteristics	
					Age	Female participants
Zhaowu Meng, 2024 (21)	Retrospective cohort	2019.09 - 2022.01	Multicenter	Canada​	60.8(mean)	50.5% (1526/3021)
Livia Archibugi, 2023 (22)	Prospective cohort	2017.06 - 2022.10	International multicenter	Italy, Spain, Sweden, Finland, Croatia	68.4{{h}} {{/h}}±{{h}} {{/h}}14.5(mean ± SD)	48.8% (561/1150)
Ping Zhu, 2023 (23)	Retrospective cohort	2014.01 - 2022.09	Single center	China	52.74 ± 9.81(mean ± SD)	50% (499/998)
Rintaro Fukuda, 2023 (24)	Retrospective cohort	2010.08 - 2020.10	Multicenter	Japan	Development: Median70(IQR 59-78)External validation:Median72(IQR 62-82)	Development: 40% (890/2224) External validation:43% (377/875)
Todd Brenner, 2025 (15)	observational analysis based on experimental data	2012.01 - 2022.09	International multicenter	USA, Netherlands, Germany, Korea, China, India	Development: 56.2(mean) External validation: NA	Development:8.6% (632/7389) External validation: NA
Ruhua Zheng, 2020 (25)	Retrospective cohort	2016.01 - 2019.12	Single center	China	Development: Age ≤60 years: 39.5%, age>60 years: 60.5%. Internal Validation Model: Age ≤60 years: 40.7%, age>60 years: 59.3%. External Validation Model: Age ≤60 years: 41.4%. Age >60 years: 58.6%.	Development: 44.4% (635/1431) Internal validation: 48.4% (296/612) External validation: 44.7% (153/342)
Youming Xu, 2024 (26)	Retrospective cohort	2017.01 - 2021.12	Multicenter	China	Development Model: 62.33 ± 15.10(mean ± SD) Internal Validation Model: 62.71 ± 15.19 (mean ± SD) External Validation Model: 64.25 ± 14.53(mean ± SD)	Development:51.8% (605/1168) Internal validation:51.76% (263/508) External validation:50.95% (107/210)
Chaoqun Yan, 2024 (27)	Retrospective cohort	2015.01 - 2023.01	Multicenter	China	Development Model: 46.67 ± 10.69 (mean ± SD) Internal Validation Model: 46.34 ± 10.37(mean ± SD) External Validation Model: NA	Development:56.57% (520/919) Internal validation:58.47% (107/183) External validation: NA
Jianhong Yao, 2023 (28)	Retrospective cohort	2019.01 - 2021.12	Single center	China​	57.25 ± 12.38(mean ± SD)	57.18% (231/404)
Shuo Wang, 2024 (29)	Retrospective cohort	2019.09 - 2023.03	Single center	China	65.27 ± 14.24(mean ± SD)	46% female (198/431)
Ma Yayun, 2023 (30)	Retrospective cohort	2017.01 - 2022.03	Multicenter	China	Development:61.2 ± 12.4(mean ± SD) External validation: 59.8 ± 13.1(mean ± SD)	Development: 47.76% (288/603) External validation:46.3% (95/205)
Mitsuru Sugimoto, 2024 (31)	Retrospective cohort	2020.11 - 2022.10	Multicenter	Japan.	Development cohort: 73.8 ± 12.7 years (mean ± SD). Validation cohort: 75.1 ± 12.5 years (mean ± SD).	38.1%
Zhifeng Fu, 2024 (32)	Retrospective cohort	2018.01 - 2022.12	Single center	China.	62.4 ± 13.2(mean ± SD)	42.7% (535/1253)
Hirokazu Saito, 2022 (33)	Retrospective cohort	2012.04 - 2020.02	Multicenter	Japan	74.3(mean)	47.1% (731/1551)
Kangjie Chen, 2024 (34)	Retrospective cohort	2019.01 - 2022.06	Multicenter	China	Development:60.3 ± 15.3(mean ± SD) External validation: 59.7 ± 14.9(mean ± SD)	Development:38% (127/341) testing cohort:44.1% (64/145) External validation:31.3% (30/96)
Chan Hyuk Park, 2022 (35)	Retrospective cohort	2015.07 - 2020.07	Multicenter	South Korea	63.5 ± 17.1 (mean ± SD)	44.3% (662/1495)
Jin-yuan Chi, 2023 (36)	Retrospective cohort	2011.10 - 2016.10	Single center	China​	60.5(mean)	60.0% (288/480)
Matthew J, 2013 (37)	Retrospective cohort	1997.01 - 2009.03	Single center	USA​	50.4(mean)	63.7% (356/559)
Kapil Kohli, 2021 (38)	Retrospective cohort	2011.03 - 2017.03	Single center	USA​	54(mean)	63.2% (170/269)
Masafumi Chiba, 2021 (39)	Retrospective cohort	2012.01 - 2019.12	Single center	Japan	Median 66.7(range24-91)	36.1% (1214/3362)
Yeon Kyung Lee, 2017 (40)	Retrospective cohort	2013.01 - 2014.08	Single center	Korea​	Median 62.3(range14-93)	40.9% (211/516)
Chan Hyuk Park, 2024 (41)	Prospective cohort y.	2020.08-2023.12	Single center	South Korea	External validation: 64.3 ± 17.8 (mean ± SD)	44.3%(493/1112)
Zhang Yan, 2023 (42)	Combined data	2010.6-2020.6	Single center.	China​	Median 61 years (IQR 50.0–72.0).	50.3%​

Among the 21 studies focused on model development, the diagnostic criteria for PEP varied. The Cotton criteria (43) were employed by eight studies (38.1%), while the Atlanta criteria (44)and the revised Atlanta criteria (45) were used by six studies (28.6%) and four studies (19%), respectively., and the Guidelines (46) were applied in one study (4.8%). Two studies (9.5%) did not follow any standardized diagnostic criteria, instead using descriptive methods. Regarding the timing of PEP onset, most studies focused on the early postoperative period. A range of <24 hours was reported in eight studies (38.1%), followed by <72 hours and <48 hours in two studies (9.5%) each. One study (4.8%) reported PEP onset within the 24–48 hours and <5-day windows. Long-term observations were less common, with one study (4.8%) observing PEP onset for <2 weeks, another (4.8%) for 24 hours to 2 weeks, and one study (4.8%) for <30 days.

### Model development and performance

Twenty-one prediction models for PEP were developed across 23 studies. Of these, nine models (9/21) included external validation sets. **Table 2** presents the prediction models, development methods, and performance metrics. Among the 21 models, 16 (16/28) used traditional Logistic Regression (LR) alone, while five models (5/21) incorporated machine learning algorithms. These machine learning methods included Gradient Boosting (GB), Random Forest (RF), Gradient-Boosted Machines (GBM), Deep Learning (DL), and Classification and Regression Tree (CART).

**Table 2:** Characteristics of the models included in the systematic review and critical appraisal for risk of bias and applicability.

Author, year	Modelling method	Sample size	Events n(%)	No predictors		EPV	Selection of candidate predictors	Selection of final predictors	Number (%) and handling of missing data	Type of validation	Performance measures
				Cand.	Final						
Zhao Wu Meng, 2024 (21)	Logistic regression(LR)​	3,021	151 (5.0)	25	8	18.9	Based on prior knowledge	Stepwise selection	n (%): NR Method: Multiple imputation	Int: Bootstrap Ext: None	Cal: Calibration plot/Slope/CITL Disc: C-Statistic/AUC graph Ov: Brier score
Livia Archibugi, 2023 (22)	Gradient Boosting (GB) and Logistic Regression (LR)​ ​	1,150	70 (6.1)	15	10	7.0	Based on medical relevance and feature importance from GB model​	All predictors included in the GBM algorithm	n (%): NR Method: Other	Int: Cross-validation Ext: None	Cal: Calibration plot Disc: C-Statistic/AUC graph Ov: Not evaluated
Ping Zhu, 2023 (23)	Logistic regression(LR)​	998	52 (5.2)	8	6	8.7	Based on univariable associations	Backward elimination	n (%): NR Method: NR	Int: Bootstrap Ext: None	Cal: Calibration plot/HL test Disc: C-Statistic/AUC graph Ov: Not evaluated
Rintaro Fukuda, 2023 (24)	Logistic regression(LR)​	Development: 2224 External validation: 875	Development: 159(7.1%) External validation: 64(7.3%)	13	5	23.8	Based on univariable associations	Stepwise selection	n (%): NR Method: NR	Int: Bootstrap Ext: Geographical	Cal: Calibration plot Disc: C-Statistic/AUC graph Ov: Not evaluated
Todd Brenner, 2025 (15)	Logistic regression(LR).Random forest (RF).Gradient-Boosted Machines (GBM)	Development: 7389 External validation: 135	Development: 632(8.6%) External validation: 14(10.4%)	20	5	89.4	Based on prior knowledge	All predictors included in the GBM algorithm	n (%): NR Method: Median imputation and k-nearest neighbors	Int: Cross-validation Ext: Completely independent	Cal: Calibration plot Disc: C-Statistic/AUC graph Ov: Not evaluated
Ruhua Zheng, 2020 (25)	Logistic regression(LR)​	Development: 1431 Internal validation:612 External validation:342	Development: 104(7.3%) Internal validation:44(7.2%) External validation:47(13.7%)	31	9	8.1	Based on univariable associations	Backward elimination	n (%): 504 (24.7) Method: excluded	Int: Cross-validation Ext: Temporal	Cal: Not evaluated Disc: C-Statistic/AUC graph Ov: Not evaluated
Youming Xu, 2024 (26)	Random Forest(RF),Logistic Regression(LR)	Development: 1168 Internal Validation:508 External validation:210	Development: 82(6.9%) Internal Validation:36(7.0%) External validation:15(7.1%)	49	8	10.3	Based on univariable associations	Stepwise selection	n (%): 371 (19.4) Method: Complete case analysis	Int: Cross-validation Ext: Geographical	Cal: Calibration plot Disc: C-Statistic/AUC graph Ov: Not evaluated
Chaoqun Yan, 2024 (27)	Logistic regression(LR)​	Development:736 Internal Validation:183 External validation:81	Development: 39(5.3%) Internal validation: 9(4.9%) External validation: NA	28	22	2.2	Based on prior knowledge and univariate analysis	LASSO selection	n (%): 37 (4.0) Method: excluded	Int: Cross-validation Ext: Geographical	Cal: Calibration plot Disc: C-Statistic/AUC graph Ov: Not evaluated
Jianhong Yao, 2023 (28)	Logistic regression(LR)​	404	41 (10.1)	7	5	8.2	Based on univariable associations	Stepwise selection	n (%): NR Method: NR	Int: Cross-validation Ext: None	Cal: Not evaluated Disc: C-Statistic/AUC graph Ov: Not evaluated
Shuo Wang, 2024 (29)	Logistic regression(LR)​	431	40 (9.3)	15	3	13.3	Based on univariable associations	Stepwise selection	n (%): NR Method: NR	Int: Bootstrap Ext: None	Cal: Calibration plot Disc: AUC graph Ov: Not evaluated
Ma Yayun, 2023 (30)	Logistic regression(LR)​	Development: 603 External validation:205	Development: 45(7.5%) External validation: 23(11.2%)	9	7	8.1	Based on univariable associations	Stepwise selection	n (%): NR Method: NR	Int: Split-sample validation. Ext: Temporal	Cal: Not evaluated Disc: AUC graph Ov: Not evaluated
Mitsuru Sugimoto, 2024 (31)	Logistic regression(LR)​	Development: 1037 Internal validation:1037	Development: 70(6.8%) Internal validation: 64(6.2%)	7	5	14.0	Based on univariable associations	Backward elimination	n (%): NR Method: excluded	Int: Split-sample validation. Ext: None	Cal: HL test Disc: C-Statistic Ov: Not evaluated
Zhifeng Fu, 2024 (32)	Logistic regression(LR)​	1,253	112 (8.9)	9	5	22.4	Based on univariable associations	Backward elimination	n (%): NR Method: Multiple imputation	Int: Cross-validation Ext: None	Cal: Calibration plot/HL test Disc: C-Statistic/AUC graph Ov: Not evaluated
Hirokazu Saito, 2022 (33)	Logistic regression(LR)​	1,551	71 (4.6)	10	5	14.2	Based on prior knowledge	Pre-specified model (no selection)	n (%): NR Method: NR	Int: None Ext: None	Cal: Not evaluated Disc: C-Statistic/AUC graph Ov: Not evaluated
Kangjie Chen, 2024 (34)	Logistic Regression (LR) with Deep Learning (DL)	Development: 341 Internal validation:145 External validation: 96	Development: 48(14.1%) testing cohort:48(33.1%) External validation: 32(33.3%)	49	6	17.2	univariate significance (p < 0.2) and machine learning	LASSO selection	n (%): NR Method: NR	Int: Cross-validation Ext: Geographical	Cal: Calibration plot/HL test Disc: C-Statistic/AUC graph Ov: Net reclassification improvement (NRI), integrated discrimination improvement (IDI)
Chan Hyuk Park, 2022 (35)	Logistic regression(LR)​	1,495	74 (4.9)	14	5	14.8	Based on univariable associations	Backward elimination	n (%): NR Method: excluded	Ext: Zhang Yan, 2024(Completely independent) Ext: Chan Hyuk Park, 2024, Geographical	Cal: HL test Disc: C-Statistic/AUC graph Ov: Not evaluated
Jin-yuan Chi, 2023 (36)	Logistic regression(LR)​	480	75 (15.6)	19	9	8.3	Based on univariable associations	Stepwise selection	n (%): NR Method: NR	Int: None Ext: None	Cal: Calibration plot Disc: C-Statistic/AUC graph Ov: Not evaluated
Matthew J, 2013 (37)	Logistic regression(LR)​	559	211 (37.7)	16	6	35.2	Based on univariable associations	Forward selection	n (%): NR Method: excluded	Int: none Ext: None	Cal: Not evaluated Disc: C-Statistic Ov: Not evaluated
Kapil Kohli, 2021 (38)	Classification and Regression Tree, CART	269	22 (8.2)	52	3	7.3	Exploratory Factor Analysis (EFA) reduced predictors	Recursive Partitioning​	n (%): NR Method: excluded	Int: None Ext: None	Cal: Not evaluated Disc: AUC graph Ov: Not evaluated
Masafumi Chiba, 2021 (39)	Logistic regression(LR)​	3,362	108 (3.2)	35	5	21.6	Based on univariable associations	Backward elimination	n (%): NR Method: Complete case analysis	Int: Bootstrap Ext: Zhang Yan2024(Completely independent)	Cal: Calibration plot/HL test Disc: C-Statistic/AUC graph Ov: Not evaluated
Yeon Kyung Lee, 2017 (40)	Logistic regression(LR)​	516	16 (3.1)	14	4	4.0	Based on univariable associations	Stepwise selection	n (%): NR Method: NR	Int: None Ext: None	Cal: Not evaluated Disc: C-Statistic/AUC graph Ov: Not evaluated

All models reported AUC values, ranging from 0.62 to 0.98 across the 21 studies included. While calibration is crucial for clinical applications, ensuring that a model’s predicted probabilities align with the actual occurrence of events, it enhances the model’s credibility and practical utility (47). Different studies have employed various calibration evaluation techniques. Among the studies included, calibration plots were used in (15, 21–24, 26, 27, 29, 32, 34, 39), as these plots provide a clear visualization of the relationship between predicted probabilities and actual outcomes. The Hosmer-Lemeshow test was utilized in (5, 23, 31, 32, 35, 39), a commonly used statistical method that assesses model fit by comparing the observed event frequencies across different predicted probability groups (47). More advanced calibration metrics, such as the Brier score, calibration slope, and CITL (48), were reported in (21), offering more detailed calibration measurements. The Brier score, in particular, is a key metric for evaluating the accuracy of predictions, considering the differences between predicted probabilities and actual outcomes. Six studies (28, 30, 33, 37, 38, 40) did not report any calibration methods, which hinders a comprehensive assessment of these models’ practical applicability. The lack of calibration evaluation in these studies raises concerns about potential biases in their clinical application.

### Factor selection

The most common method for selecting candidate predictors was univariable analysis, used in 13 studies (61.9%). Four studies (19.0%) relied on prior knowledge. Two studies combined feature importance from the Gradient Boosting (GB) model with univariable significance (p < 0.2) and machine learning techniques. One study used exploratory factor analysis (EFA) for factor selection.

For final predictor selection, stepwise selection was the most common, applied in 8 studies (38.1%). Backward elimination was used in 6 studies (28.6%), and LASSO selection in 2 studies (9.5%). The GBM algorithm (with all predictors) was used in 2 studies (9.5%). Forward selection and recursive partitioning appeared in 1 study (4.8%) each, and a pre-specified model in 1 study (4.8%).

### Factors included in prediction models

These factors can be categorized based on their modifiability and clinical relevance: modifiable factors include difficult cannulation (13 models), operator experience (4 models), history of bile duct stones (4 model), and precut sphincterotomy (4 models), which can be addressed during the procedure to guide preventive strategies.

Disease-related factors, such as a history of pancreatitis (6 models), pancreatic duct injection/visualization (7 models), and pancreatic sphincterotomy (7 models), are important for prediction but cannot be altered during surgery.

Demographic factors, such as female sex (9 models) and age (7 models), are based on patient characteristics and are typically known before the procedure.

**Figure 2:**
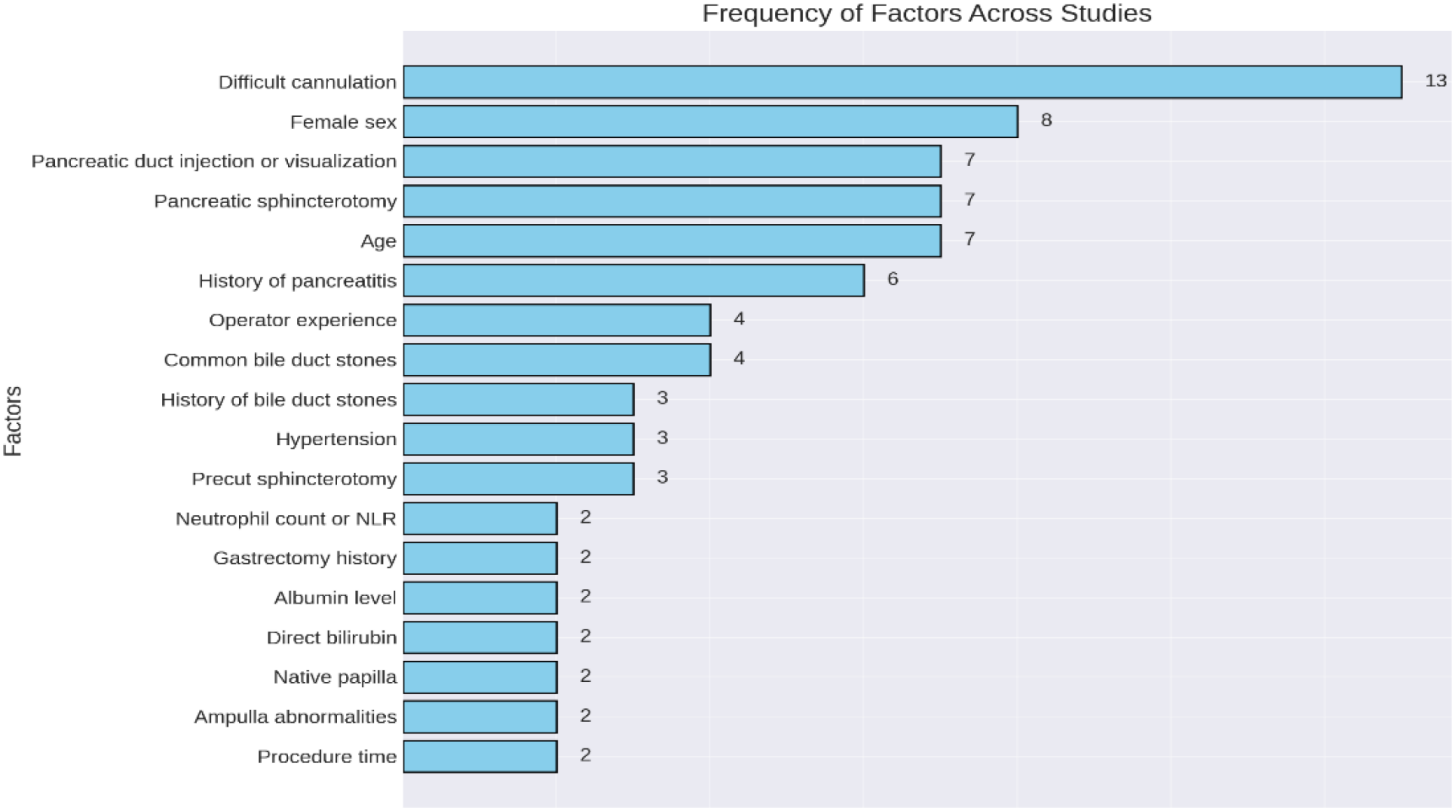
Risk factors included in PEP prediction models.

Less common predictors include hypertension (3 models), neutrophil count (2 models), hypoalbuminemia (2 models), and direct bilirubin (2 models), which play a role in prediction but appear less frequently in the models (**Figure 2**).

### Risk of bias and applicability assessment

**Figure 3:**
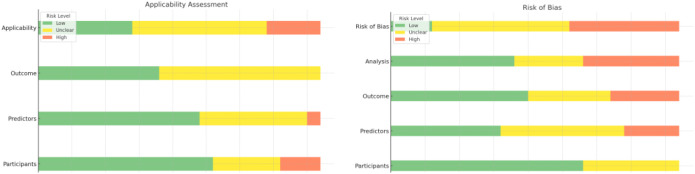
Risk- of- bias (ROB) assessment using the PROBAST tool.

The bias risk and applicability assessments of the 21 model development studies are summarized in **Table 3**. Three studies (25, 26, 39) (14.3%) were rated as having low overall bias risk, while 10 studies (15, 22, 24, 27, 30, 32–36) (47.6%) had an unclear bias risk, and 8 studies (21, 23, 28, 29, 31, 37, 38, 40) (38.1%) had a high risk of bias (**Figure 3**). Based on the PROBAST tool assessment, the sources of bias primarily included unclear definitions of predictor variables, inadequate sample sizes and EPV (events per variable) that did not meet recommended values, insufficient explanation of missing data handling, absence of external validation sets, and inadequate model calibration and performance interpretation.

**Table 3:** Risk of bias and applicability assessment.

Author, year	Risk of Bias				Applicability			Overall	
	1. Participants	2. Predictors	3. Outcome	4. Analysis	1. Participants	2. Predictors	3. Outcome	Risk of Bias	Applicability
Zhao Wu Meng, 2024 (21)	+	?	–	–	?	+	?	–	?
Livia Archibugi, 2023 (22)	?	?	?	?	+	?	?	?	?
Ping Zhu, 2023 (23)	?	–	?	–	–	?	+	–	–
Rintaro Fukuda, 2023 (24)	+	?	+	+	+	+	+	?	+
Todd Brenner, 2025 (15)	+	+	?	+	+	+	+	?	+
Ruhua Zheng, 2020 (25)	+	+	+	+	?	+	?	+	?
Youming Xu, 2024 (26)	+	+	+	+	+	+	+	+	+
Chaoqun Yan, 2024 (27)	+	?	+	+	+	+	+	?	+
Jianhong Yao, 2023 (28)	?	–	–	–	?	–	?	–	–
Shuo Wang, 2024 (29)	?	–	–	–	?	?	?	–	?
Ma Yayun, 2023 (30)	+	?	+	?	+	+	?	?	?
Mitsuru Sugimoto, 2024 (31)	+	+	?	–	+	+	+	–	+
Zhifeng Fu, 2024 (32)	?	?	+	?	–	?	?	?	–
Hirokazu Saito, 2022 (33)	+	?	+	+	+	?	+	?	?
Kangjie Chen, 2024 (34)	+	?	+	+	+	+	+	?	+
Chan Hyuk Park, 2022 (35)	+	+	?	+	+	+	+	?	+
Jin-yuan Chi, 2023 (36)	+	+	+	?	+	+	?	?	?
Matthew J, 2013 (37)	+	?	–	?	?	?	?	–	?
Kapil Kohli, 2021 (38)	?	+	?	–	+	+	?	–	?
Masafumi Chiba, 2021 (39)	+	+	+	+	+	?	?	+	?
Yeon Kyung Lee, 2017 (40)	?	–	–	–	–	?	?	–	–

Regarding applicability, 7 studies (15, 24, 26, 27, 31, 34, 35) (33.3%) demonstrated good performance in terms of the representativeness of the study population, clinical relevance of predictive factors, and the application of outcome measures. For 10 studies (21, 23, 25, 29, 30, 33, 36–39)(47.6%), the applicability was unclear, while 4 studies (23, 28, 32, 40) (19.0%) raised significant concerns regarding applicability. The EPV range for these four studies was between 2.2 and 8.3, below the recommended minimum of 10. These studies had sample sizes ranging from 269 to 559 participants, which were insufficient for stable model development and validation. None of these studies performed external validation, nor did they provide detailed explanations for missing data handling or report model calibration plots or relevant statistical tests.

Four studies (15, 25, 26, 39) demonstrated comprehensive performance across all evaluation domains. In these 4 studies, the recommended EPV was ≥10. Ruhua Zheng (2020) and Todd Brenner (2025) employed multiple imputation methods, while Youming Xu (2024) utilized a complete-case analysis. All four studies reported external validation results and calibration performance. The model complexity was well matched to the sample size in each of these studies.

### Meta-analysis and subgroup analysis

**Figure 4:**
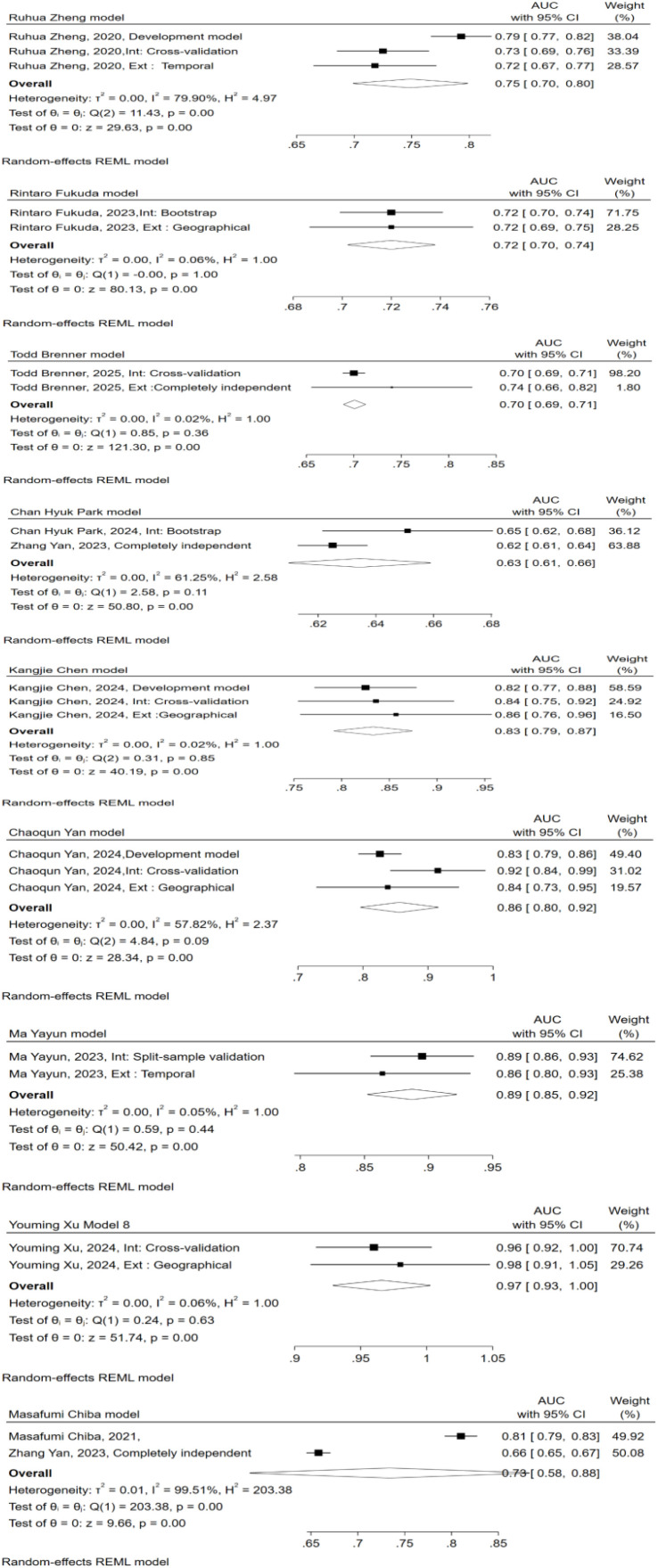
Forest plot with the area under the receiver operating characteristics curve for studies that externally validated the PEP prediction model.

The meta-analysis assessed the performance of nine externally validated predictive models for PEP. As shown in **Figure 4**, The AUC values ranged from 0.63 to 0.97, with the Youming Xu (2024) model demonstrating the highest performance (AUC{{h}} {{/h}}={{h}} {{/h}}0.97 [0.93, 1.00]). Six models (15, 24, 26, 27, 30, 34) showed heterogeneity below 10%, indicating that their performance was relatively consistent across different validation sets. Three models (25, 39, 41) showed high heterogeneity, with I² values greater than 50%, including Chan Hyuk Park (2024) (I² = 61.25%), Ruhua Zheng (2020) (I² = 79.90%), and Masafumi Chiba (2021) (I² = 99.51%). The results of the z-test for all models were statistically significant (p < 0.05), indicating robust predictive capability during external validation.

**Figure 5:**
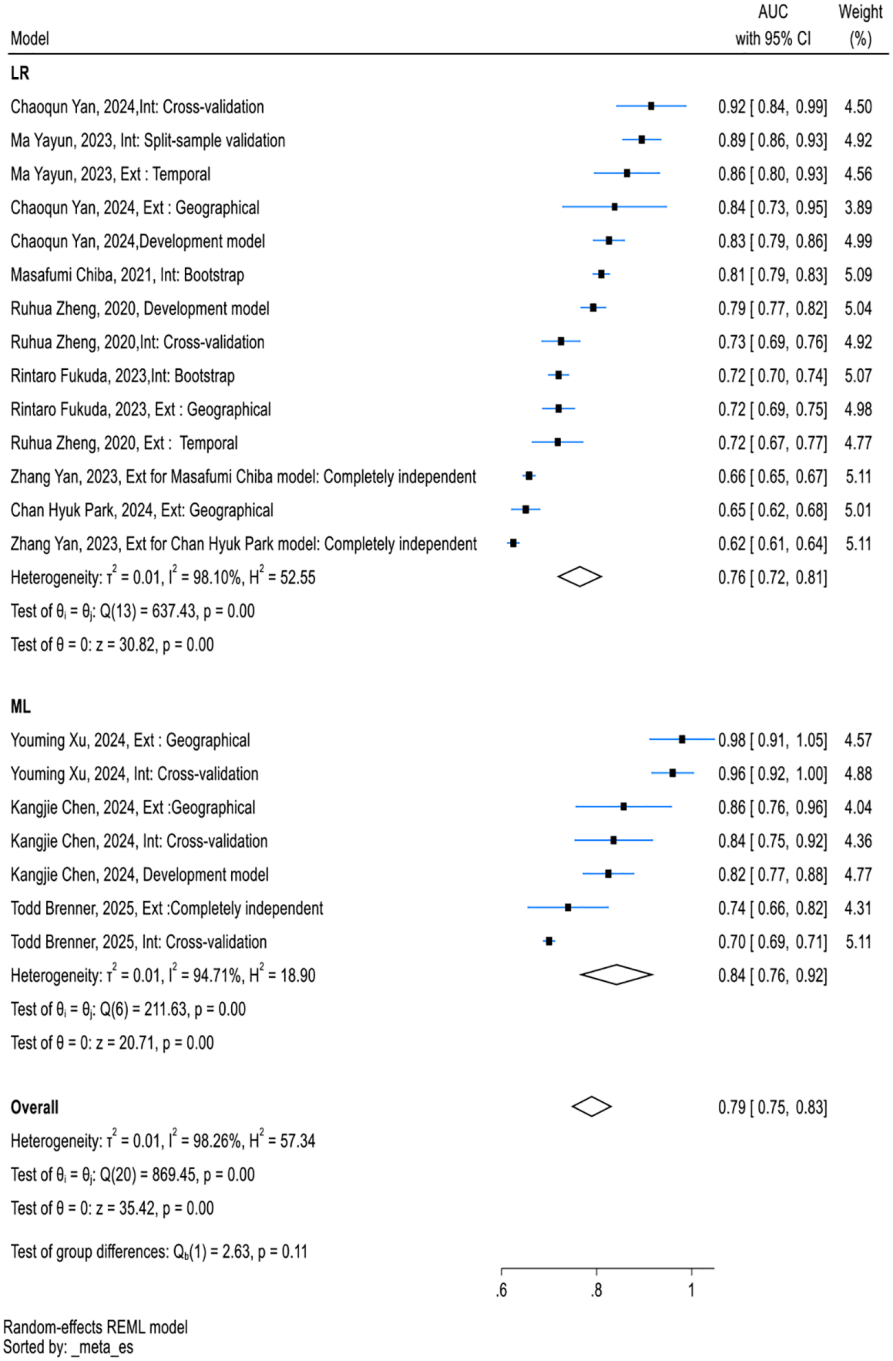
Forest plot for subgroup analysis of traditional models and machine learning models in externally validated PEP models.

**Figure 6:**
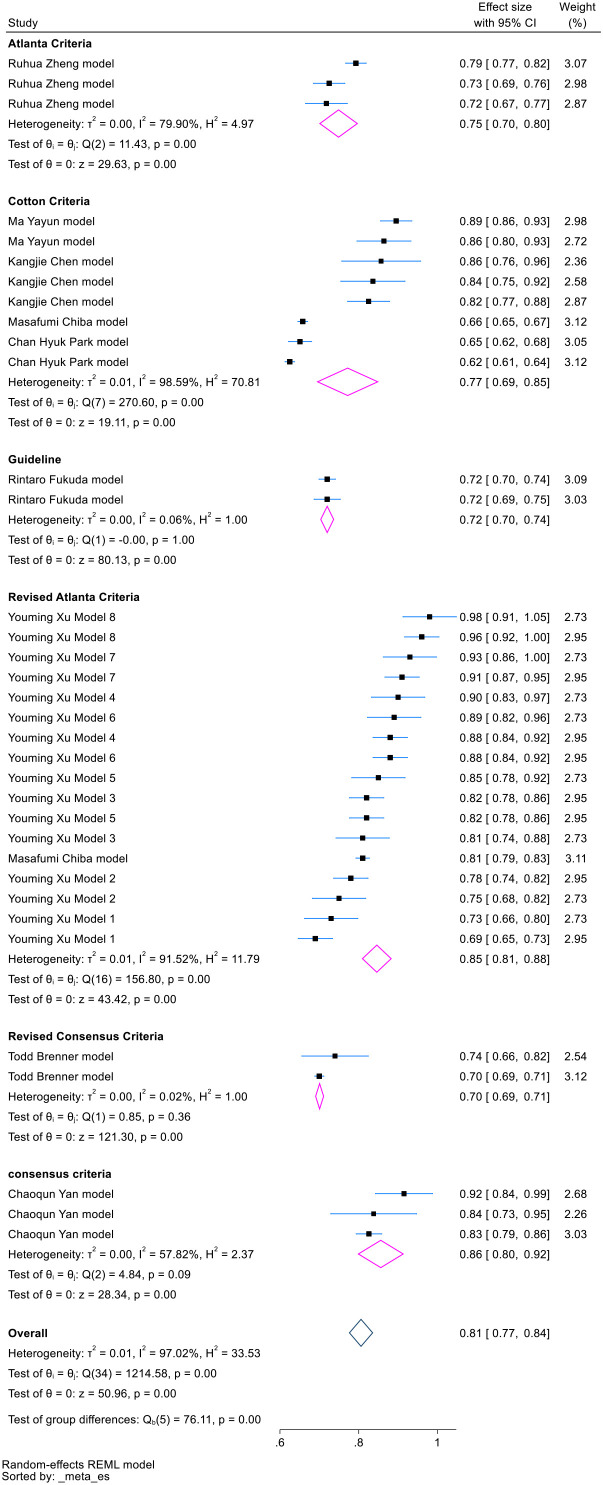
Forest plot for subgroup analysis of PEP diagnostic criteria in externally validated PEP models.

We performed subgroup analysis based on model type and PEP diagnostic criteria. Both traditional models and machine learning models exhibited considerable heterogeneity across the subgroups (**Figure 5**). However, categorizing by PEP diagnostic criteria led to a notable reduction in heterogeneity (**Figure 6**).

## Discussion

This systematic review included 23 studies and 21 predictive models for PEP. one study recalibrated an existing model, and one validated two prior models. Nine models underwent external validation, Nomograms and scoring systems were the most commonly used tools for PEP risk prediction. However, the diagnostic criteria and time of outcome occurrence varied significantly across studies, which poses a challenge to model comparison analysis.

Among the 21 model development studies, 38.1% used the Cotton criteria, 28.6% used the Atlanta classification, and 9.5% used the revised Atlanta classification. Another 9.5% used descriptive methods without clear definitions for hospitalization duration or imaging requirements. According to Smeets et al. (49), the revised Atlanta criteria demonstrate superior performance in assessing PEP severity and predicting mortality, with sensitivity, specificity, and positive predictive value (PPV) of 100%, 98%, and 58%, respectively, compared to the consensus criteria (55% sensitivity, 72% specificity, and 5% PPV). Importantly, the revised criteria depend on standardized imaging protocols that may be influenced by technical and operator variability (50, 51). Despite these limitations, the revised criteria have been consistently shown to be superior for defining PEP severity across multiple studies (52, 53), while the consensus criteria remain valuable for patient-centered care (49). Through subgroup analysis, we found that the PEP diagnostic criteria may be a source of heterogeneity in model performance.

Based on the meta-analysis, Xu’s model showed the best performance with an AUC of 0.96 in internal validation and 0.98 in external validation. The model achieved an accuracy of 92.77%, sensitivity of 86.11%, and specificity of 93.28%. It was constructed using a stepwise approach and incorporated baseline characteristics, procedural factors, preventive strategies, and imaging features. Imaging data played a crucial role in risk assessment, which significantly enhanced accuracy by 7.03% (from 85.74% to 92.77%), sensitivity by 2.78% (from 83.33% to 86.11%), and specificity by 7.36% (from 85.92% to 93.28%) (26). Although externally validated, Broader validation in more diverse patient populations is needed to enhance its clinical applicability, Studies have shown that external validation can significantly improve model accuracy by up to 10% (54), sensitivity by 15%, and specificity by 12% (55), highlighting its importance in ensuring model generalizability.

In comparing traditional models with machine learning (ML) models (**Figure 5**), we found that ML models had a pooled AUC of 0.84, performing well but exhibiting substantial heterogeneity (I² = 94.71%), especially in studies with smaller sample sizes or lower data quality. The pooled AUC for logistic regression (LR)-based models was 0.76, with similarly high heterogeneity (I² = 98.10%). Sensitivity analysis revealed that the heterogeneity of traditional models did not significantly decrease, likely due to differences in sample sizes, diagnostic criteria for PEP, and varying measurement times.

Interestingly, Xu’s model performed excellently in the overall analysis but was identified as a major source of heterogeneity in subgroup analysis. Excluding Xu’s model significantly reduced the heterogeneity of ML models (I² = 84.61%). This may be due to the inclusion of multimodal features, such as imaging data, which increases the risk of overfitting (56, 57). The high-dimensional nature of imaging data can lead to overfitting by memorizing specific details from the training set, reducing the model’s ability to generalize to new datasets (58, 59). Therefore, while Xu’s model showed excellent performance in internal and external validation, its performance in heterogeneity analysis suggests potential overfitting issues associated with complex models. To mitigate such risks, techniques like cross-validation and penalization are commonly employed in high-dimensional models to enhance generalizability and control overfitting.

In this study, machine learning (ML) models achieved a higher pooled AUC than logistic regression (0.84 vs. 0.76). XU’s model achieved an AUC of 0.98 in external validation, demonstrating excellent discriminatory performance, this may enable earlier identification of high-risk patients, informing preventive measures such as NSAID administration or intraoperative management. Nevertheless, the clinical utility of ML models remains uncertain. BISAP, the most widely used severity score in acute pancreatitis, incorporates five variables available within 24 hours: BUN >25 mg/dL, altered mental status, SIRS, age >60 years, and pleural effusion has an AUC of 0.72-0.82, with sensitivity of 65-70% and specificity of 75-80% (60, 61), balancing simplicity and accuracy. While ML models often yield higher AUCs, A systematic review found no consistent advantage of ML or deep learning over traditional scoring systems in practical clinical use (62). They require more inputs, involve complex processing, and lack interpretability, limiting bedside use. Xu’s model uses imaging features, making it reliant on imaging data, such imaging-based models face considerable challenges in real-world barriers including inadequate infrastructure, incomplete data annotation, inter-observer variability, and heterogeneous imaging acquisition protocols, especially in resource-constrained settings. Financial and institutional constraints also hinder implementation (63).

To enhance clinical applicability, high-performance models should undergo repeated external validation across different time periods, institutions, and countries to ensure generalizability (64). Once performance is consistently stable, implementation should begin on a small scale in selected hospitals. Leveraging frameworks such as DEPLOYR (65), Models can be embedded into existing EHR workflows with real-time data capture and intuitive output displays. Silent deployment, continuous monitoring, and user feedback enable prospective evaluation, providing a solid foundation for broader implementation.

## Limitations and strengths

The lack of standardized diagnostic criteria for PEP limits the ability to compare models. Future studies should focus on creating uniform diagnostic standards. Overfitting is another issue in machine learning models, particularly with multimodal or imaging data. Many models, including Xu’s, lack independent prospective validation. Future studies should test models in different centers and populations to confirm their effectiveness.

This study presents several innovations and advantages. We employed standardized templates based on the CHARMS and PROBAST checklists to comprehensively assess the performance, predictive factors, factor selection methods, events per variable (EPV), and model construction techniques across various predictive models. We identified the best-performing PEP prediction models and highlighted potential overfitting issues in some. Additionally, we conducted subgroup analysis comparing traditional and machine learning models and found that the revised Atlanta criteria significantly reduced heterogeneity, improving model applicability and consistency, especially in externally validated models. Finally, we explored the potential clinical application of high-performance models, proposing methods such as multimodal model integration, including imaging data, to enhance predictive accuracy and facilitate clinical implementation.

## Conclusion

This systematic review demonstrates that while existing PEP prediction models perform reasonably well, significant differences in diagnostic criteria, data quality, and external validation remain. Future research should address these issues, including the adoption of standardized PEP definitions, standardized imaging assessment methods, and reducing overfitting in machine learning models.
