# Supplementary material for: Predictive models for post-ERCP pancreatitis: a systematic review and meta-analysis
Source: Front Gastroenterol (Lausanne). 2026 Mar 5;4:1629698. doi: 10.3389/fgstr.2025.1629698 (PMC13000349; doi:10.3389/fgstr.2025.1629698)
Supplement: Supplementary file 1 [file Table1.docx]

**Appendix I: Search strategies**

**Search strategies used for finding research articles about prediction models for post-ERCP pancreatitis. Number of retrieved articles is given in the right-hand column.**

**PubMed**

| #1 | ERCP[Title/Abstract] OR "endoscopic retrograde cholangiopancreatography"[Title/Abstract] | 17947 |
| --- | --- | --- |
| #2 | "post-ERCP pancreatitis"[Title/Abstract] OR "PEP"[Title/Abstract] OR "ERCP complications"[Title/Abstract] | 11224 |
| #3 | "prediction model"[Title/Abstract] OR "predictive model"[Title/Abstract] OR "predict*"[Title/Abstract] OR "prognostic model"[Title/Abstract] OR "risk assessment"[Title/Abstract] OR "risk stratification"[Title/Abstract] OR "clinical prediction tool"[Title/Abstract] | 2403270 |
| #4 | "sensitivity"[Title/Abstract] OR "specificity"[Title/Abstract] OR "AUC"[Title/Abstract] OR "area under the curve"[Title/Abstract] OR "ROC curve"[Title/Abstract] OR "calibration"[Title/Abstract] OR "discrimination"[Title/Abstract] OR "decision curve analysis"[Title/Abstract] | 1735389 |
| #5 | #1 AND #2 AND (#3 OR #4 ) | 271 |

**Web of Science**

| #1 | TS= ("ERCP" OR "endoscopic retrograde cholangiopancreatography") | 14660 |
| --- | --- | --- |
| #2 | TS= ("post-ERCP pancreat*" OR "PEP" OR "pancreatitis after ERCP" OR "ERCP complications") | 10545 |
| #3 | TS= ("prediction model" OR "predictive model" OR "predict*" OR "prognostic model" OR "risk assessment" OR "risk stratification" OR "clinical prediction tool") | 3545056 |
| #4 | TS= ("sensitivity" OR "specificity" OR "AUC" OR "area under the curve" OR "ROC curve" OR "calibration" OR "discrimination" OR "decision curve analysis") | 2039245 |
| #5 | #1 AND #2 AND (#3 OR #4 ) | 288 |

**Embase**

| #1 | 'endoscopic retrograde cholangiopancreatography'/exp OR ERCP | 56822 |
| --- | --- | --- |
| #2 | 'post-ERCP pancreat*' OR PEP OR 'pancreat* after ERCP' OR 'ERCP complications' | 22595 |
| #3 | 'prediction model' OR 'predictive model' OR 'predict*' OR 'prognostic model' OR 'risk assessment' OR 'risk stratification' OR 'clinical prediction tool' | 3615409 |
| #4 | 'sensitivity' OR 'specificity' OR 'AUC' OR 'area under curve' OR 'roc curve'/exp OR 'calibration' OR 'discrimination' OR 'decision curve analysis' | 3021256 |
| #5 | #1 AND #2 AND (#3 OR #4 ) | 960 |

**The Cochrane Library**

| #1 | "ERCP" OR "endoscopic retrograde cholangiopancreatography" IN Title Abstract Keyword | 2495 |
| --- | --- | --- |
| #2 | "post-ERCP pancreatitis" OR "PEP" OR "pancreatitis after ERCP" OR "ERCP complications" IN Title Abstract Keyword | 2464 |
| #3 | "prediction model" OR "predictive model" OR "predict*" OR "prognostic model" OR "risk assessment" OR "risk stratification" OR "clinical prediction tool" IN Title Abstract Keyword | 64167 |
| #4 | "sensitivity" OR "specificity" OR "AUC" OR "area under the curve" OR "ROC curve" OR "calibration" OR "discrimination" OR "decision curve analysis" IN Title Abstract Keyword | 131755 |
| #5 | #1 AND #2 AND (#3 OR #4 ) | 93 |

**CNKI**

| #1 | SU= "ERCP" OR "内镜逆行胰胆管造影" | 9443 |
| --- | --- | --- |
| #2 | SU="ERCP术后胰腺炎" OR "PEP" OR "胰腺炎" OR "ERCP术后并发症" | 824 |
| #3 | SU= "预测模型" OR "风险预测" OR "预后模型" OR "风险评估" OR "风险分层" OR "临床预测工具"OR "预测因子" OR "预测结果" OR "预测性能" | 2812 |
| #4 | SU= "AUC" OR "ROC曲线下面积" OR "校准" OR "区分能力" OR "决策曲线分析" | 1211 |
| #5 | #1 AND #2 AND (#3 OR #4) | 55 |

**TOTAL FOUND: 1667**
